# Data on hydrocarbons in sediment samples, and its body burden levels in tissues of *Anomalocardia flexuosa* from toxicity testing

**DOI:** 10.1016/j.dib.2020.105889

**Published:** 2020-06-18

**Authors:** Lucas Buruaem Moreira, Silvio Tarou Sasaki, Satie Taniguchi, Rubens Cesar Lopes Figueira, Márcia Caruso Bícego, Leticia Veras Costa-Lotufo, Denis Moledo Souza Abessa

**Affiliations:** a Instituto de Ciências do Mar, Universidade Federal do Ceará, Fortaleza, Brazil; b Núcleo de Estudos em Poluição e Ecotoxicologia Aquática, UNESP São Vicente, Brazil; c Instituto Oceanográfico, Universidade de São Paulo, São Paulo, Brazil

**Keywords:** Bioaccumulation, Environmental monitoring, Linear alkylbenzenes, Marine pollution, Polycyclic aromatic hydrocarbons, Sublethal effects, Tropical environments, Toxicity testing

## Abstract

The analysis of hydrocarbons in terms of individual compounds is relevant to understand the origin and source of these substances, as well as its distribution in environmental compartments, including sediments and biota. Hydrocarbons concentrations were determined in sediments and in whole-body soft tissues of the tropical clam *Anomalocardia flexuosa* in sediment toxicity testing using samples of Mucuripe bay (Ceará State, NE Brazil) collected in 2011 during dredging events [1]. Data of target compounds included aliphatic (AHs) and aromatic hydrocarbons (PAHs), and linear alkylbenzenes (LABs). AHs compounds were determined on gas chromatography with flame ionization detector (GC-FID), while PAHs and LABs were determined on gas chromatography coupled to a mass spectrometer (GC/MS) in a selected ion mode (SIM). The potential of this dataset is baseline information on hydrocarbons contamination in sediments from a semi-arid region and the bioaccumulation of organic contaminants in marine organisms that can be used as models in ecotoxicological studies.

**Specifications Table**

**Table:** 

**Subject**	Environmental Science
**Specific subject area**	Sediment contamination of hydrocarbons and bioaccumulation assessment
**Type of data**	Table
**How data were acquired**	Sediment sampling: van Veen grabs; whole-body tissues of clam obtained from sediment toxicity testing. Hydrocarbons were measured by gas chromatography with flame ionization detector (GC-FID) model 6890 from Agilent Technologies, and gas chromatography coupled to a 5973 N mass spectrometer (GC/MS) in a selected ion mode (SIM) model 6890 Agilent Technologies.
**Data format**	Raw
**Parameters for data collection**	Surface sediment samples were collected in two campaigns, during the intense dredging (Survey 1) and after the end of operations (Survey 2) at Mucuripe bay (Ceará State, NE Brazil) in sites influenced by contamination sources, harbour activities and dredging, including the reference site Icapuí (Ceará State, NE Brazil). Clams were manually collected during low tide in muddy banks at reference site, transported to laboratory and exposed to sediment samples by means of a whole-sediment toxicity protocol. Sediment samples and whole-body soft tissues samples were analyzed for contents of hydrocarbons.
**Description of data collection**	Samples were collected using a van Veen grab covering the top 2 cm of the surface layer. Clams were collected and exposed to sediments for 28 days, with subsamples of whole-body soft tissues obtained at different times (0, 7, 14, 21, and 28 days) for bioaccumulation analysis. Analysis of compounds in tissues and sediments included aliphatic hydrocarbons by gas chromatography with flame ionization detector (GC-FID), and polycyclic aromatic hydrocarbons and linear alkylbenzenes by gas chromatography coupled to a mass spectrometer (GC/MS) in a selected ion mode (SIM)
**Data source location**	City/Region: cities of Fortaleza and Icapuí, Ceará state, Brazil. Latitude and longitude: Icapuí: 4°40′54.7″S 37°20′13.9″W; MD1: 3°42′35.4″S 38°28′29.3″W; MD2: 3°42′41.7″S 38°28′57.2″W; MD3: 3°42′06.0″S 38°29′55.1″W
**Data accessibility**	With the article.
**Related research article**	Moreira et al. 2020. Biomarkers responses of the clam *Anomalocardia flexuosa* in sediment toxicity bioassays using dredged materials from a semi-arid coastal system. Heliyon 6, e04030. https://doi.org/10.1016/j.heliyon.2020.e04030

**Value of the Data**

• The dataset comprises a detailed profile of hydrocarbons in the sediments, dredging materials, and clams of the species *A. flexuosa* in experiments performed in one of the most important harbour of the Northeast Region of Brazil.
• The dataset can assist researchers on ecotoxicology and marine pollution, policymakers, and stakeholders to understand the sediment contamination by hydrocarbons along the coastal zone of a continental-scale country such as Brazil, where differences in sedimentology that regulate bioavailability are observed.
• Data on individual compounds can provide information on potential sources and origin of hydrocarbons, which are important to determine the contaminants of environmental concern related to harbour activities in Tropical regions.
• Data on individual compounds can assist the pollution control strategies and other management actions
• The dataset contributes to the development of site-specific sediment quality values, which are originated from the integrated analysis of sediment chemistry matched with biological effects associated with contamination levels.

## 1 Data description

Sediment samples collected in Mucuripe bay during dredging activities (survey 1, January 2011) and at the end of operations (survey 2, July 2011) were analyzed for hydrocarbon contamination in September 2012. A profile of concentrations of twenty-six AHs from n-C17 to n-C35, including pristane and phytane is provided in Table 1. Table 2 presents data of thirty-three PAHs measured in sediment samples, while the profiles of twenty-six LABs are shown in Table 3. Sediment toxicity testing with *A. flexuosa* exposed to samples for 28 days with records at 7 days intervals generated profiles of twenty-six AHs from n-C17 to n-C35, including pristane and phytane in whole-body tissues for survey 1 (during dredging) in Table 4, and for survey 2 (after dredging) in Table 5. Profiles of thirty-nine PAHs were produced for tissues of clams from experiments for survey 1 in Table 6, and for survey 2, the profiles are reported in Table 7. Concentrations of twenty-six LABs analyzed in *A. flexuosa* from survey 1 exposures are given in Table 8, and the results of survey 2 are given in Table 9.

**Table 1:** Concentrations of aliphatic hydrocarbons (AHs) measured in sediments from Mucuripe Bay. Values expressed in µg g^−1^ (dry weight). Reference site located at Icapuí.

Compound	Reference	Survey 1			Survey 2		
		MD1	MD2	MD3	MD1	MD2	MD3
n-C12	<0.003	0.03	0.00	0.00	0.01	<0.003	<0.003
n-C13	0.003	0.10	0.00	0.01	0.02	0.00	<0.001
n-C14	<0.002	0.08	0.01	0.00	0.06	0.01	0.01
n-C15	0.015	0.09	0.01	<0.004	0.06	0.01	0.01
n-C16	<0.001	0.07	<0.001	<0.001	0.02	<0.001	<0.001
n-C17	0.047	0.21	0.01	<0.005	0.12	0.01	0.01
Pristane	<0.008	0.19	<0.008	0.01	0.19	<0.008	<0.008
n-C18	0.004	0.25	0.00	<0.002	0.11	<0.002	<0.002
Phytane	0.004	0.30	0.01	0.01	0.26	<0.002	<0.002
n-C19	<0.005	0.13	<0.005	<0.005	0.09	<0.005	<0.005
n-C20	<0.008	0.02	<0.008	<0.008	<0.008	<0.008	<0.008
n-C21	<0.011	0.08	<0.011	<0.011	0.04	<0.011	<0.011
n-C22	0.006	0.13	<0.003	<0.003	0.06	<0.003	0.00
n-C23	<0.006	0.17	<0.006	<0.006	0.13	<0.006	0.01
n-C24	<0.006	0.10	<0.006	<0.006	0.03	<0.006	0.01
n-C25	<0.027	0.14	<0.027	<0.027	0.09	<0.027	<0.027
n-C26	<0.007	0.10	0.01	<0.007	0.07	<0.007	0.01
n-C27	<0.034	0.08	<0.034	<0.034	0.08	<0.034	<0.034
n-C28	<0.034	0.12	<0.034	<0.034	0.11	<0.034	<0.034
n-C29	<0.028	0.18	0.03	<0.028	0.19	<0.028	<0.028
n-C30	<0.028	0.38	<0.028	<0.028	0.28	<0.028	<0.028
n-C31	<0.026	0.31	0.04	<0.026	0.30	<0.026	<0.026
n-C32	<0.026	0.24	<0.026	<0.026	0.18	<0.026	<0.026
n-C33	<0.012	0.02	0.03	<0.012	0.19	<0.012	<0.012
n-C34	<0.012	<0.012	<0.012	<0.012	0.02	<0.012	<0.012
n-C35	<0.012	0.03	<0.012	<0.012	0.04	<0.012	<0.012

**Table 2:** Concentrations of polycyclic aromatic hydrocarbons (PAHs) measured in sediments from Mucuripe Bay. Values expressed in ng g^−1^ (dry weight). Reference site located at Icapuí.

Compound	Reference	Survey 1			Survey 2		
		MD1	MD2	MD3	MD1	MD2	MD3
Naphthalene	<1.60	4.7	<1.60	<1.60	9.59	<1.60	<1.60
Methylnaphthalenes	<1.30	12.52	<1.30	<1.30	15.1	<1.30	<1.30
Biphenyl	<1.30	1.4	<1.30	<1.30	1.47	<1.30	<1.30
Ethylnaphthalenes	<2.60	4.16	<2.60	<2.60	4.44	<2.60	<2.60
Dimethylnaphthalenes	<2.60	44.97	<2.60	<2.60	37.89	<2.60	<2.60
Acenaphthylene	<3.70	<3.70	<3.70	<3.70	<3.70	<3.70	<3.70
Acenaphthene	<1.30	1.42	<1.30	<1.30	<1.30	<1.30	<1.30
Trimethylnaphthalenes	<1.30	78.7	<1.30	<1.30	59.32	<1.30	<1.30
Fluorene	<1.30	6.51	<1.30	<1.30	4.03	<1.30	<1.30
Dibenzothiophene	<1.30	4.13	<1.30	<1.30	21.39	<1.30	<1.30
Phenanthrene	<2.60	19.5	<2.60	<2.60	15.72	<2.60	<2.60
Anthracene	<1.10	3.99	<1.10	<1.10	3.24	<1.10	<1.10
Methylphenanthrenes	<2.20	65.8	<2.20	<2.20	76.11	<2.20	<2.20
Fluoranthene	<1.30	34.92	<1.30	<1.30	24.66	<1.30	<1.30
Pyrene	<1.30	42.19	<1.30	<1.30	35.43	<1.30	<1.30
Methylfluoranthenes	<1.30	55.59	<1.30	<1.30	28.38	<1.30	<1.30
Retene	<1.30	20.93	<1.30	<1.30	22.25	<1.30	<1.30
Methylpyrenes	<1.30	99.44	<1.30	<1.30	66.43	<1.30	<1.30
Benzo(c)phenanthrene	<1.20	5.77	<1.20	<1.20	2.1	<1.20	<1.20
Benzo(a)anthracene	<1.20	26.82	<1.20	<1.20	11.63	<1.20	<1.20
Chrysene	<1.20	82.6	<1.20	<1.20	41.14	<1.20	<1.20
Methylchrysene	<1.20	163.07	<1.20	<1.20	87.1	<1.20	<1.20
Benzo(b)fluoranthene	<1.30	42.56	<1.30	<1.30	19.04	<1.30	<1.30
Benzo(j)fluoranthene	<1.30	32	<1.30	<1.30	2.94	<1.30	<1.30
Benzo(k)fluoranthene	<1.30	32.15	<1.30	<1.30	4.13	<1.30	<1.30
Benzo(e)pyrene	<1.30	77.41	<1.30	<1.30	28.11	<1.30	<1.30
Benzo(a)pyrene	<1.10	51	<1.10	<1.10	13.43	<1.10	<1.10
Perylene	<1.20	33.69	2.96	<1.20	18.72	<1.20	<1.20
Indeno[1,2,3-c,d]pyrene	<1.00	44.17	<1.00	<1.00	10.65	<1.00	<1.00
Dibenzo(a,h)anthracene	<1.00	19.13	<1.00	<1.00	6.16	<1.00	<1.00
Benzo(b)chrysene	<1.10	10.05	<1.10	<1.10	3.1	<1.10	<1.10
Benzo(ghi)perylene	<1.20	33.11	<1.20	<1.20	14.53	<1.20	<1.20
Coronene	<1.20	6.03	<1.20	<1.20	2.65	<1.20	<1.20

**Table 3:** Concentrations of linear alkylbenzenes (LABs) measured in sediments from Mucuripe Bay. Values expressed in ng g^−1^ (dry weight). Reference site located at Icapuí.

Compound	Reference	Survey 1			Survey 2		
		MD1	MD2	MD3	MD1	MD2	MD3
5-C_10_-LAB	<1.68	<1.68	<1.68	<1.68	<1.68	<1.68	<1.68
4-C_10_-LAB	<1.68	<1.68	<1.68	<1.68	<1.68	<1.68	<1.68
3-C_10_-LAB	<1.68	<1.68	<1.68	<1.68	<1.68	<1.68	<1.68
2-C_10_-LAB	<1.68	<1.68	<1.68	<1.68	<1.68	<1.68	<1.68
6-C_11_-LAB	<0.85	<0.85	<0.85	<0.85	4.65	<0.85	<0.85
5-C_11_-LAB	<0.85	<0.85	<0.85	<0.85	7.31	<0.85	<0.85
4-C_11_-LAB	<0.85	<0.85	<0.85	<0.85	5.39	<0.85	<0.85
3-C_11_-LAB	<0.85	<0.85	<0.85	<0.85	3.57	<0.85	<0.85
2-C_11_-LAB	<0.85	<0.85	<0.85	<0.85	8.02	<0.85	<0.85
6-C_12_-LAB	<0.99	<0.99	<0.99	<0.99	9.65	<0.99	<0.99
5-C_12_-LAB	<0.99	<0.99	<0.99	<0.99	7.13	<0.99	<0.99
4-C_12_-LAB	<0.99	<0.99	<0.99	<0.99	5.2	<0.99	<0.99
3-C_12_-LAB	<0.99	<0.99	<0.99	<0.99	2.91	<0.99	<0.99
2-C_12_-LAB	<0.99	<0.99	<0.99	<0.99	4.37	<0.99	<0.99
7-C_13_-LAB	<1.31	<1.31	<1.31	<1.31	5.275	<1.31	<1.31
6-C_13_-LAB	<1.31	<1.31	<1.31	<1.31	5.275	<1.31	<1.31
5-C_13_-LAB	<1.31	<1.31	<1.31	<1.31	6.64	<1.31	<1.31
4-C_13_-LAB	<1.31	<1.31	<1.31	<1.31	3.71	<1.31	<1.31
3-C_13_-LAB	<1.31	<1.31	<1.31	<1.31	3.42	<1.31	<1.31
2-C_13_-LAB	<1.31	<1.31	<1.31	<1.31	9.1	<1.31	<1.31
7-C_14_-LAB	<0.85	<0.85	<0.85	<0.85	<0.85	<0.85	<0.85
6-C_14_-LAB	<0.85	<0.85	<0.85	<0.85	<0.85	<0.85	<0.85
5-C_14_-LAB	<0.85	<0.85	<0.85	<0.85	<0.85	<0.85	<0.85
4-C_14_-LAB	<0.85	<0.85	<0.85	<0.85	<0.85	<0.85	<0.85
3-C_14_-LAB	<0.85	<0.85	<0.85	<0.85	<0.85	<0.85	<0.85
2-C_14_-LAB	<0.85	<0.85	<0.85	<0.85	<0.85	<0.85	<0.85

**Table 4:** Concentrations of aliphatic hydrocarbons (AHs) in whole-body tissues of *Anomalocardia flexuosa* exposed to sediments from Mucuripe Bay and reference site located at Icapuí (Survey 1). Values expressed in µg g^−1^ (dry weight).

Sample	Control	Reference				MD1				MD2					MD3		
Time	Day 0	Day 7	Day 14	Day 21	Day 28	Day 7	Day 14	Day 21	Day 28	Day 7	Day 14	Day 21	Day 28	Day 7	Day 14	Day 21	Day 28
n-C12	<0.164	<0.164	<0.164	<0.164	<0.164	<0.164	<0.164	<0.164	<0.164	<0.164	<0.164	<0.164	<0.164	<0.164	<0.164	<0.164	<0.164
n-C13	<0.133	<0.133	<0.133	<0.133	<0.133	<0.133	<0.133	<0.133	<0.133	<0.133	<0.133	<0.133	<0.133	<0.133	<0.133	<0.133	<0.133
n-C14	<0.117	<0.117	<0.117	<0.117	<0.117	<0.117	<0.117	<0.117	<0.117	<0.117	<0.117	<0.117	<0.117	<0.117	<0.117	<0.117	<0.117
n-C15	<0.099	<0.099	<0.099	<0.099	<0.099	<0.099	<0.099	<0.099	<0.099	<0.099	<0.099	<0.099	<0.099	<0.099	<0.099	<0.099	<0.099
n-C16	<0.096	<0.096	<0.096	<0.096	<0.096	<0.096	<0.096	<0.096	<0.096	<0.096	<0.096	<0.096	<0.096	<0.096	<0.096	<0.096	<0.096
n-C17	<0.091	0.333	<0.091	<0.091	<0.091	0.155	0.137	0.214	0.115	0.110	<0.091	0.220	0.124	0.105	0.092	<0.091	<0.091
Pristane	<0.093	<0.093	<0.093	<0.093	<0.093	<0.093	<0.093	<0.093	<0.093	<0.093	<0.093	<0.093	<0.093	<0.093	<0.093	<0.093	<0.093
n-C18	<0.087	<0.087	<0.087	<0.087	<0.087	<0.087	<0.087	<0.087	<0.087	<0.087	<0.087	<0.087	<0.087	<0.087	<0.087	<0.087	<0.087
Phytane	<0.085	<0.085	<0.085	<0.085	<0.085	<0.085	<0.085	<0.085	<0.085	<0.085	<0.085	<0.085	<0.085	<0.085	<0.085	<0.085	<0.085
n-C19	<0.083	<0.083	<0.083	<0.083	<0.083	<0.083	<0.083	<0.083	<0.083	<0.083	<0.083	<0.083	<0.083	<0.083	<0.083	<0.083	<0.083
n-C20	<0.075	<0.075	<0.075	<0.075	<0.075	<0.075	<0.075	<0.075	<0.075	<0.075	<0.075	<0.075	<0.075	<0.075	<0.075	<0.075	<0.075
n-C21	<0.076	0.118	<0.076	<0.076	0.094	<0.076	<0.076	0.151	<0.076	0.114	<0.076	0.115	0.119	0.080	<0.076	0.085	<0.076
n-C22	0.099	0.333	0.184	0.256	0.319	0.259	0.199	0.375	0.227	0.399	0.195	0.368	0.336	0.296	0.260	0.230	0.210
n-C23	0.082	0.310	0.174	0.280	0.316	0.320	0.135	0.252	0.221	0.390	0.256	0.382	0.391	0.273	0.247	0.268	0.280
n-C24	0.114	0.353	0.231	0.386	0.350	0.501	0.164	0.221	0.260	0.474	0.310	0.435	0.405	0.309	0.273	0.315	0.333
n-C25	<0.074	0.231	0.171	0.294	0.227	0.392	0.111	0.108	0.154	0.320	0.221	0.263	0.227	0.180	0.164	0.218	0.199
n-C26	<0.081	0.116	0.085	0.136	0.122	0.187	<0.081	<0.081	<0.081	0.141	0.110	0.104	0.104	<0.081	<0.081	0.104	0.086
n-C27	<0.094	<0.094	<0.094	<0.094	<0.094	<0.094	<0.094	<0.094	<0.094	<0.094	<0.094	<0.094	<0.094	<0.094	<0.094	<0.094	<0.094
n-C28	<0.120	<0.120	<0.120	<0.120	<0.120	<0.120	<0.120	<0.120	<0.120	<0.120	<0.120	<0.120	<0.120	<0.120	<0.120	<0.120	<0.120
n-C29	<0.134	<0.134	<0.134	<0.134	<0.134	<0.134	<0.134	<0.134	<0.134	<0.134	<0.134	<0.134	<0.134	<0.134	<0.134	<0.134	<0.134
n-C30	<0.152	<0.152	<0.152	<0.152	<0.152	<0.152	<0.152	<0.152	<0.152	<0.152	<0.152	<0.152	<0.152	<0.152	<0.152	<0.152	<0.152
n-C31	<0.134	<0.134	<0.134	<0.134	<0.134	<0.134	<0.134	<0.134	<0.134	<0.134	<0.134	<0.134	<0.134	<0.134	<0.134	<0.134	<0.134
n-C32	<0.131	<0.131	<0.131	<0.131	<0.131	<0.131	<0.131	<0.131	<0.131	<0.131	<0.131	<0.131	<0.131	<0.131	<0.131	<0.131	<0.131
n-C33	<0.110	<0.110	<0.110	<0.110	<0.110	<0.110	<0.110	<0.110	<0.110	<0.110	<0.110	<0.110	<0.110	<0.110	<0.110	<0.110	<0.110
n-C34	<0.134	<0.134	<0.134	<0.134	<0.134	<0.134	<0.134	<0.134	<0.134	<0.134	<0.134	<0.134	<0.134	<0.134	<0.134	<0.134	<0.134
n-C35	<0.134	<0.134	<0.134	<0.134	<0.134	<0.134	<0.134	<0.134	<0.134	<0.134	<0.134	<0.134	<0.134	<0.134	<0.134	<0.134	<0.134

**Table 5:** Concentrations of aliphatic hydrocarbons (AHs) in whole-body tissues of *Anomalocardia flexuosa* exposed to sediments from Mucuripe Bay and reference site located at Icapuí (Survey 2). Values expressed in µg g^−1^ (dry weight).

Sample	Control	Reference				MD1				MD2				MD3			
Time	Day 0	Day 7	Day 14	Day 21	Day 28	Day 7	Day 14	Day 21	Day 28	Day 7	Day 14	Day 21	Day 28	Day 7	Day 14	Day 21	Day 28
n-C12	<0.164	<0.164	<0.164	<0.164	<0.164	<0.164	<0.164	<0.164	<0.164	<0.164	<0.164	<0.164	<0.164	<0.164	<0.164	<0.164	<0.164
n-C13	<0.133	<0.133	<0.133	<0.133	<0.133	<0.133	<0.133	<0.133	<0.133	<0.133	<0.133	<0.133	<0.133	<0.133	<0.133	<0.133	<0.133
n-C14	<0.117	<0.117	<0.117	<0.117	<0.117	<0.117	<0.117	<0.117	<0.117	<0.117	<0.117	<0.117	<0.117	<0.117	<0.117	<0.117	<0.117
n-C15	<0.099	<0.099	<0.099	<0.099	<0.099	<0.099	<0.099	<0.099	<0.099	<0.099	<0.099	<0.099	<0.099	<0.099	<0.099	<0.099	<0.099
n-C16	<0.096	<0.096	<0.096	<0.096	<0.096	<0.096	<0.096	<0.096	<0.096	<0.096	<0.096	<0.096	<0.096	<0.096	<0.096	<0.096	<0.096
n-C17	<0.091	<0.091	<0.091	<0.091	<0.091	<0.091	<0.091	<0.091	<0.091	<0.091	<0.091	<0.091	<0.091	<0.091	<0.091	<0.091	<0.091
Pristane	<0.093	<0.093	<0.093	<0.093	<0.093	<0.093	<0.093	<0.093	<0.093	<0.093	<0.093	<0.093	<0.093	<0.093	<0.093	<0.093	<0.093
n-C18	<0.087	<0.087	<0.087	<0.087	<0.087	<0.087	<0.087	<0.087	<0.087	<0.087	<0.087	<0.087	<0.087	<0.087	<0.087	<0.087	<0.087
Phytane	<0.085	<0.085	<0.085	<0.085	<0.085	<0.085	<0.085	<0.085	<0.085	<0.085	<0.085	<0.085	<0.085	<0.085	<0.085	<0.085	<0.085
n-C19	<0.083	<0.083	<0.083	<0.083	<0.083	<0.083	<0.083	<0.083	<0.083	<0.083	<0.083	<0.083	<0.083	<0.083	<0.083	<0.083	<0.083
n-C20	<0.075	<0.075	<0.075	<0.075	<0.075	<0.075	<0.075	<0.075	<0.075	<0.075	<0.075	<0.075	<0.075	<0.075	<0.075	<0.075	<0.075
n-C21	<0.076	<0.076	0.082	0.077	<0.076	<0.076	0.084	<0.076	<0.076	0.114	<0.076	<0.076	<0.076	0.098	<0.076	<0.076	<0.076
n-C22	0.170	0.206	0.302	0.269	0.221	0.153	0.299	0.136	0.142	0.346	0.227	0.141	0.243	0.297	0.235	0.090	0.219
n-C23	0.228	0.222	0.337	0.434	0.197	0.185	0.292	0.146	0.226	0.371	0.335	0.153	0.379	0.431	0.243	0.142	0.225
n-C24	0.299	0.239	0.403	0.616	0.192	0.206	0.304	0.165	0.351	0.398	0.502	0.172	0.553	0.560	0.280	0.240	0.267
n-C25	0.186	0.121	0.242	0.402	0.079	0.126	0.171	0.084	0.248	0.239	0.348	0.093	0.364	0.363	0.161	0.174	0.170
n-C26	<0.081	<0.081	0.086	0.152	<0.081	<0.081	<0.081	<0.081	0.089	0.094	0.146	<0.081	0.148	0.156	<0.081	<0.081	<0.081
n-C27	<0.094	0.110	<0.094	<0.094	<0.094	<0.094	<0.094	<0.094	<0.094	<0.094	<0.094	<0.094	<0.094	<0.094	<0.094	<0.094	<0.094
n-C28	<0.120	<0.120	<0.120	<0.120	<0.120	<0.120	<0.120	<0.120	<0.120	<0.120	<0.120	<0.120	<0.120	<0.120	<0.120	<0.120	<0.120
n-C29	<0.134	<0.134	<0.134	<0.134	<0.134	<0.134	<0.134	<0.134	<0.134	<0.134	<0.134	<0.134	<0.134	<0.134	<0.134	<0.134	<0.134
n-C30	<0.152	<0.152	<0.152	<0.152	<0.152	<0.152	<0.152	<0.152	<0.152	<0.152	<0.152	<0.152	<0.152	<0.152	<0.152	<0.152	<0.152
n-C31	<0.134	<0.134	<0.134	<0.134	<0.134	<0.134	<0.134	<0.134	<0.134	<0.134	<0.134	<0.134	<0.134	<0.134	<0.134	<0.134	<0.134
n-C32	<0.131	<0.131	<0.131	<0.131	<0.131	<0.131	<0.131	<0.131	<0.131	<0.131	<0.131	<0.131	<0.131	<0.131	<0.131	<0.131	<0.131
n-C33	<0.110	<0.110	<0.110	<0.110	<0.110	<0.110	<0.110	<0.110	<0.110	<0.110	<0.110	<0.110	<0.110	<0.110	<0.110	<0.110	<0.110
n-C34	<0.134	<0.134	<0.134	<0.134	<0.134	<0.134	<0.134	<0.134	<0.134	<0.134	<0.134	<0.134	<0.134	<0.134	<0.134	<0.134	<0.134
n-C35	<0.134	<0.134	<0.134	<0.134	<0.134	<0.134	<0.134	<0.134	<0.134	<0.134	<0.134	<0.134	<0.134	<0.134	<0.134	<0.134	<0.134

**Table 6:** Concentrations of polycyclic aromatic hydrocarbons (PAHs) in whole-body tissues of *Anomalocardia flexuosa* exposed to sediments from Mucuripe Bay and reference site located at Icapuí (Survey 1). Values expressed in ng g^−1^ (dry weight).

Sample	Control	Reference				MD1				MD2				MD3		
Time	Day 0	Day 7	Day 14	Day 21	Day 28	Day 7	Day 14	Day 21	Day 28	Day 7	Day 14	Day 21	Day 28	Day 7	Day 14	Day 21
Naphthalene	6.47	<5.80	<5.80	8.56	10.50	13.70	<5.80	<5.80	<5.80	11.53	21.04	<5.80	7.36	7.80	28.46	8.16
Methylnaphthalenes	6.22	8.45	7.88	9.89	8.52	13.51	9.60	10.16	6.96	9.99	23.82	6.68	12.85	9.63	28.35	10.75
Biphenyl	<4.75	<4.75	<4.75	<4.75	<4.75	<4.75	<4.75	<4.75	<4.75	<4.75	<4.75	<4.75	<4.75	<4.75	<4.75	<4.75
Ethylnaphthalenes	<4.71	<4.71	<4.71	<4.71	<4.71	<4.71	<4.71	<4.71	<4.71	<4.71	<4.71	<4.71	<4.71	<4.71	<4.71	<4.71
Dimethylnaphthalenes	17.51	12.98	11.44	18.67	14.42	25.33	17.65	19.74	15.92	14.18	41.32	16.46	19.30	15.72	48.02	17.68
Acenaphthylene	<3.83	<3.83	<3.83	<3.83	<3.83	<3.83	<3.83	<3.83	<3.83	<3.83	<3.83	<3.83	<3.83	<3.83	<3.83	<3.83
Acenaphthene	<5.87	<5.87	<5.87	<5.87	<5.87	<5.87	<5.87	<5.87	<5.87	<5.87	<5.87	<5.87	<5.87	<5.87	<5.87	<5.87
Trimethylnaphthalenes	8.53	<7.27	<7.27	<7.27	<7.27	22.23	<7.27	<7.27	<7.27	9.54	29.31	7.94	<7.27	8.23	<7.27	<7.27
Fluorene	<5.16	<5.16	<5.16	<5.16	<5.16	<5.16	<5.16	<5.16	<5.16	<5.16	<5.16	<5.16	<5.16	<5.16	<5.16	<5.16
Methyl fluorene	<10.3	<10.3	<10.3	<10.3	<10.3	<10.3	<10.3	<10.3	<10.3	<10.3	<10.3	<10.3	<10.3	<10.3	<10.3	<10.3
Dibenzothiophene	<1.41	<1.41	<1.41	<1.41	<1.41	<1.41	<1.41	<1.41	<1.41	<1.41	<1.41	<1.41	<1.41	<1.41	<1.41	<1.41
Phenanthrene	6.82	<3.48	<3.48	5.68	7.31	11.88	13.52	7.31	6.15	8.90	11.98	6.63	4.12	7.82	6.98	8.65
Anthracene	<3.46	<3.46	<3.46	<3.46	<3.46	<3.46	<3.46	<3.46	<3.46	<3.46	<3.46	<3.46	<3.46	<3.46	<3.46	<3.46
Dimethyl fluorene	<10.3	<10.3	<10.3	<10.3	<10.3	<10.3	<10.3	<10.3	<10.3	<10.3	<10.3	<10.3	<10.3	<10.3	<10.3	<10.3
Methyl dibenzothiophene	<2.81	<2.81	<2.81	<2.81	<2.81	<2.81	<2.81	<2.81	<2.81	<2.81	<2.81	<2.81	<2.81	<2.81	<2.81	<2.81
Methylphenanthrenes	<5.85	<5.85	<5.85	8.30	<5.85	<5.85	<5.85	23.51	16.73	<5.85	14.00	<5.85	<5.85	6.24	<5.85	<5.85
Dimethyl dibenzothiophene	<2.81	<2.81	<2.81	<2.81	<2.81	<2.81	<2.81	<2.81	<2.81	<2.81	3.97	<2.81	<2.81	<2.81	<2.81	<2.81
Dimethyl phenanthrenes	<5.85	<5.85	<5.85	<5.85	<5.85	<5.85	<5.85	<5.85	<5.85	<5.85	6.22	<5.85	<5.85	<5.85	<5.85	<5.85
Fluoranthene	25.39	10.99	6.65	18.07	24.62	28.30	67.01	61.10	76.59	46.77	21.83	36.03	63.79	35.16	25.53	30.63
Pyrene	75.26	27.70	23.46	65.84	79.83	102.56	208.51	197.89	278.17	170.44	79.22	113.21	226.32	99.87	83.49	100.03
Methylfluoranthenes	<5.08	<5.08	<5.08	<5.08	<5.08	<5.08	<5.08	<5.08	<5.08	<5.08	<5.08	<5.08	<5.08	<5.08	<5.08	<5.08
Retene	48.77	26.93	35.32	<5.08	53.42	35.16	45.92	106.95	55.35	92.24	11.37	76.24	42.87	50.55	31.56	51.30
Methylpyrenes	<5.08	<5.08	<5.08	<5.08	<5.08	11.34	14.85	11.36	5.87	5.63	5.41	<5.08	<5.08	<5.08	<5.08	6.80
Benzo(c)phenanthrene	<5.08	<5.08	<5.08	<5.08	<5.08	<5.08	<5.08	<5.08	<5.08	<5.08	<5.08	<5.08	<5.08	<5.08	<5.08	<5.08
Benzo(a)anthracene	<3.71	<3.71	<3.71	<3.71	<3.71	<3.71	<3.71	<3.71	<3.71	<3.71	<3.71	<3.71	<3.71	<3.71	<3.71	<3.71
Chrysene	<3.52	<3.52	<3.52	<3.52	<3.52	7.09	12.26	7.95	6.40	<3.52	<3.52	<3.52	<3.52	<3.52	<3.52	9.81
Methylchrysene	<7.04	<7.04	<7.04	<7.04	<7.04	<7.04	<7.04	<7.04	<7.04	<7.04	<7.04	<7.04	<7.04	<7.04	<7.04	<7.04
Dimethylchrysene	<7.04	<7.04	<7.04	<7.04	<7.04	<7.04	<7.04	<7.04	<7.04	<7.04	<7.04	<7.04	<7.04	<7.04	<7.04	<7.04
Benzo(b)fluoranthene	<2.31	<2.31	<2.31	<2.31	<2.31	<2.31	<2.31	<2.31	<2.31	<2.31	<2.31	<2.31	<2.31	<2.31	<2.31	<2.31
Benzo(j)fluoranthene	<2.53	<2.53	<2.53	<2.53	<2.53	<2.53	<2.53	<2.53	<2.53	<2.53	<2.53	<2.53	<2.53	<2.53	<2.53	<2.53
Benzo(k)fluoranthene	<2.53	<2.53	<2.53	<2.53	<2.53	<2.53	<2.53	<2.53	<2.53	<2.53	<2.53	<2.53	<2.53	<2.53	<2.53	<2.53
Benzo(e)pyrene	<2.40	<2.40	<2.40	<2.40	<2.40	<2.40	<2.40	<2.40	<2.40	<2.40	<2.40	<2.40	<2.40	<2.40	<2.40	2.43
Benzo(a)pyrene	<2.56	<2.56	<2.56	<2.56	<2.56	<2.56	<2.56	<2.56	<2.56	<2.56	<2.56	<2.56	<2.56	<2.56	<2.56	<2.56
Perylene	<2.03	<2.03	<2.03	<2.03	<2.03	<2.03	<2.03	<2.03	<2.03	<2.03	<2.03	<2.03	<2.03	<2.03	<2.03	<2.03
Indeno[1,2,3-c,d]pyrene	<1.21	<1.21	<1.21	<1.21	<1.21	<1.21	<1.21	<1.21	<1.21	<1.21	<1.21	<1.21	<1.21	<1.21	<1.21	<1.21
Dibenzo(a,h)anthracene	<1.14	<1.14	<1.14	<1.14	<1.14	<1.14	<1.14	<1.14	<1.14	<1.14	<1.14	<1.14	<1.14	<1.14	<1.14	<1.14
Benzo(b)chrysene	<1.14	<1.14	<1.14	<1.14	<1.14	<1.14	<1.14	<1.14	<1.14	<1.14	<1.14	<1.14	<1.14	<1.14	<1.14	<1.14
Benzo(ghi)perylene	<1.58	<1.58	<1.58	<1.58	<1.58	<1.58	<1.58	<1.58	<1.58	<1.58	<1.58	<1.58	<1.58	<1.58	<1.58	<1.58
Coronene	<1.58	<1.58	<1.58	<1.58	<1.58	<1.58	<1.58	<1.58	<1.58	<1.58	<1.58	<1.58	<1.58	<1.58	<1.58	<1.58

**Table 7:** Concentrations of polycyclic aromatic hydrocarbons (PAHs) in whole-body tissues of *Anomalocardia flexuosa* exposed to sediments from Mucuripe Bay and reference site located at Icapuí (Survey 1). Values expressed in ng g^−1^ (dry weight).

Sample	Control	Reference				MD1				MD2				MD3			
Time	Day 0	Day 7	Day 14	Day 21	Day 28	Day 7	Day 14	Day 21	Day 28	Day 7	Day 14	Day 21	Day 28	Day 7	Day 14	Day 21	Day 28
Naphthalene	12.44	8.22	11.90	9.15	13.60	159.16	151.17	115.79	20.45	6.09	159.21	138.64	130.91	10.54	153.36	16.42	11.73
Methylnaphthalenes	14.97	12.11	9.64	10.55	13.76	56.90	14.09	15.20	20.32	10.60	13.43	20.52	46.14	15.14	13.72	12.86	11.20
Biphenyl	<4.75	<4.75	<4.75	<4.75	<4.75	12.83	5.23	<4.75	<4.75	<4.75	<4.75	4.85	10.38	<4.75	<4.75	<4.75	<4.75
Ethylnaphthalenes	<4.71	<4.71	<4.71	<4.71	<4.71	<4.71	<4.71	<4.71	<4.71	<4.71	<4.71	<4.71	<4.71	<4.71	<4.71	<4.71	<4.71
Dimethylnaphthalenes	20.89	23.11	20.51	16.83	23.02	56.68	<9.25	12.95	37.47	15.95	15.42	26.57	55.29	25.82	10.60	19.86	15.65
Acenaphthylene	<3.83	<3.83	<3.83	<3.83	<3.83	<3.83	18.09	15.49	<3.83	<3.83	18.35	14.54	<3.83	<3.83	18.06	<3.83	<3.83
Acenaphthene	<5.87	<5.87	<5.87	<5.87	<5.87	<5.87	<5.87	<5.87	<5.87	<5.87	<5.87	<5.87	<5.87	<5.87	<5.87	<5.87	<5.87
Trimethylnaphthalenes	<7.27	13.68	<7.27	<7.27	7.76	10.41	<7.27	<7.27	10.49	<7.27	<7.27	<7.27	12.82	<7.27	<7.27	<7.27	<7.27
Fluorene	<5.16	<5.16	<5.16	<5.16	<5.16	<5.16	<5.16	<5.16	<5.16	<5.16	<5.16	<5.16	<5.16	<5.16	<5.16	<5.16	<5.16
Methyl fluorene	<10.3	<10.3	<10.3	<10.3	<10.3	<10.3	<10.3	<10.3	<10.3	<10.3	<10.3	<10.3	<10.3	<10.3	<10.3	<10.3	<10.3
Dibenzothiophene	<1.41	<1.41	<1.41	<1.41	<1.41	<1.41	<1.41	<1.41	<1.41	<1.41	<1.41	<1.41	<1.41	<1.41	<1.41	<1.41	<1.41
Phenanthrene	4.75	6.92	<3.48	<3.48	5.97	10.26	16.22	17.22	4.61	4.80	21.04	11.08	13.89	<3.48	21.84	3.59	<3.48
Anthracene	<3.46	<3.46	<3.46	<3.46	<3.46	<3.46	<3.46	<3.46	<3.46	<3.46	<3.46	<3.46	<3.46	<3.46	<3.46	<3.46	<3.46
Dimethyl fluorene	<10.3	<10.3	<10.3	<10.3	<10.3	<10.3	<10.3	<10.3	<10.3	<10.3	<10.3	<10.3	<10.3	<10.3	<10.3	<10.3	<10.3
Methyl dibenzothiophene	<2.81	<2.81	<2.81	<2.81	<2.81	<2.81	<2.81	<2.81	<2.81	<2.81	<2.81	<2.81	<2.81	<2.81	<2.81	<2.81	<2.81
Methylphenanthrenes	<5.85	<5.85	<5.85	<5.85	<5.85	6.68	<5.85	<5.85	<5.85	<5.85	<5.85	<5.85	<5.85	<5.85	<5.85	<5.85	<5.85
Dimethyl dibenzothiophene	<2.81	<2.81	<2.81	<2.81	<2.81	<2.81	<2.81	<2.81	<2.81	<2.81	<2.81	<2.81	<2.81	<2.81	<2.81	<2.81	<2.81
Dimethyl phenanthrenes	<5.85	<5.85	<5.85	<5.85	<5.85	<5.85	<5.85	<5.85	<5.85	<5.85	<5.85	<5.85	<5.85	<5.85	<5.85	<5.85	<5.85
Fluoranthene	20.47	18.78	18.01	28.41	22.07	16.56	22.27	34.54	19.58	19.84	46.93	20.32	24.75	19.30	30.19	20.07	17.06
Pyrene	51.22	54.72	54.38	81.94	60.72	48.43	46.93	78.64	64.98	55.16	118.42	51.09	57.46	59.14	60.83	58.87	49.12
Methylfluoranthenes	<5.08	<5.08	<5.08	<5.08	<5.08	<5.08	<5.08	<5.08	<5.08	<5.08	<5.08	<5.08	<5.08	<5.08	<5.08	<5.08	<5.08
Retene	8.15	59.35	22.44	30.62	7.19	62.61	<5.08	<5.08	83.85	48.23	<5.08	41.82	49.64	78.78	<5.08	5.31	39.77
Methylpyrenes	<5.08	<5.08	6.77	<5.08	<5.08	5.80	5.79	<5.08	15.57	<5.08	6.07	<5.08	<5.08	<5.08	<5.08	<5.08	<5.08
Benzo(c)phenanthrene	<5.08	<5.08	<5.08	<5.08	<5.08	<5.08	<5.08	<5.08	<5.08	<5.08	<5.08	<5.08	<5.08	<5.08	<5.08	<5.08	<5.08
Benzo(a)anthracene	<3.71	<3.71	<3.71	<3.71	<3.71	<3.71	<3.71	<3.71	<3.71	<3.71	<3.71	<3.71	<3.71	<3.71	<3.71	<3.71	<3.71
Chrysene	<3.52	<3.52	8.64	<3.52	<3.52	4.49	10.99	3.95	13.79	<3.52	5.86	<3.52	<3.52	<3.52	<3.52	<3.52	<3.52
Methylchrysene	<7.04	<7.04	<7.04	<7.04	<7.04	<7.04	<7.04	<7.04	<7.04	<7.04	<7.04	<7.04	<7.04	<7.04	<7.04	<7.04	<7.04
Dimethylchrysene	<7.04	<7.04	<7.04	<7.04	<7.04	<7.04	<7.04	<7.04	<7.04	<7.04	<7.04	<7.04	<7.04	<7.04	<7.04	<7.04	<7.04
Benzo(b)fluoranthene	<2.31	<2.31	<2.31	<2.31	<2.31	<2.31	<2.31	<2.31	<2.31	<2.31	<2.31	<2.31	<2.31	<2.31	<2.31	<2.31	<2.31
Benzo(j)fluoranthene	<2.53	<2.53	<2.53	<2.53	<2.53	<2.53	<2.53	<2.53	<2.53	<2.53	<2.53	<2.53	<2.53	<2.53	<2.53	<2.53	<2.53
Benzo(k)fluoranthene	<2.53	<2.53	<2.53	<2.53	<2.53	<2.53	<2.53	<2.53	<2.53	<2.53	<2.53	<2.53	<2.53	<2.53	<2.53	<2.53	<2.53
Benzo(e)pyrene	<2.40	<2.40	<2.40	<2.40	<2.40	<2.40	<2.40	<2.40	<2.40	<2.40	<2.40	<2.40	<2.40	<2.40	<2.40	<2.40	<2.40
Benzo(a)pyrene	<2.56	<2.56	<2.56	<2.56	<2.56	<2.56	<2.56	<2.56	<2.56	<2.56	<2.56	<2.56	<2.56	<2.56	<2.56	<2.56	<2.56
Perylene	<2.03	<2.03	<2.03	<2.03	<2.03	<2.03	<2.03	<2.03	<2.03	<2.03	<2.03	<2.03	<2.03	<2.03	<2.03	<2.03	<2.03
Indeno[1,2,3-c,d]pyrene	<1.21	<1.21	<1.21	<1.21	<1.21	<1.21	<1.21	<1.21	<1.21	<1.21	<1.21	<1.21	<1.21	<1.21	<1.21	<1.21	<1.21
Dibenzo(a,h)anthracene	<1.14	<1.14	<1.14	<1.14	<1.14	<1.14	<1.14	<1.14	<1.14	<1.14	<1.14	<1.14	<1.14	<1.14	<1.14	<1.14	<1.14
Benzo(b)chrysene	<1.14	<1.14	<1.14	<1.14	<1.14	<1.14	<1.14	<1.14	<1.14	<1.14	<1.14	<1.14	<1.14	<1.14	<1.14	<1.14	<1.14
Benzo(ghi)perylene	<1.58	<1.58	<1.58	<1.58	<1.58	<1.58	<1.58	<1.58	<1.58	<1.58	<1.58	<1.58	<1.58	<1.58	<1.58	<1.58	<1.58
Coronene	<1.58	<1.58	<1.58	<1.58	<1.58	<1.58	<1.58	<1.58	<1.58	<1.58	<1.58	<1.58	<1.58	<1.58	<1.58	<1.58	<1.58

**Table 8:** Concentrations of Linear alkylbenzenes (LABs) in whole-body tissues of *Anomalocardia flexuosa* exposed to sediments from Mucuripe Bay and reference site located at Icapuí (Survey 1). Values expressed in ng g^−1^ (dry weight).

Sample	Control	Reference				MD1				MD2				MD3			
Exposure time	Day 0	Day 7	Day 14	Day 21	Day 28	Day 7	Day 14	Day 21	Day 28	Day 7	Day 14	Day 21	Day 28	Day 7	Day 14	Day 21	Day 28
5-C_10_ LAB	12.7	13.8	19.5	17.2	15.4	19.7	20.6	14.2	21.5	15.8	26.9	9.8	17.1	24.9	19.9	18.2	17.0
4-C_10_-LAB	11.0	10.6	16.8	13.1	12.6	16.6	16.9	11.1	17.0	13.1	22.4	7.9	13.1	19.4	14.6	14.7	14.1
3-C_10_-LAB	9.4	8.5	14.1	12.6	10.3	14.5	15.9	8.0	16.5	11.2	19.3	6.8	11.9	17.5	11.9	11.2	12.7
2-C_10_-LAB	12.5	11.5	20.3	18.8	13.2	24.8	26.6	13.4	25.1	19.0	27.8	9.6	18.5	26.4	15.0	17.8	21.2
6-C_11_-LAB	18.1	14.3	29.4	25.7	19.2	32.5	35.2	20.4	35.1	27.1	42.3	14.8	28.3	40.4	22.4	22.8	27.2
5-C_11_-LAB	30.5	22.5	48.6	45.9	30.6	57.8	63.1	33.9	64.2	43.6	68.1	23.6	47.0	66.2	35.3	37.9	50.9
4-C_11_-LAB	22.3	16.4	36.5	34.3	22.2	44.5	49.1	26.1	49.2	33.9	52.0	18.1	35.2	49.9	26.8	28.2	38.4
3-C_11_-LAB	15.9	11.6	27.0	26.6	15.8	33.8	36.9	19.4	39.6	25.7	37.5	14.1	26.6	36.2	19.6	20.7	28.8
2-C_11_-LAB	16.4	17.4	33.3	34.4	17.6	46.4	53.6	39.9	52.6	34.3	48.3	25.0	33.5	48.2	33.2	25.7	39.0
6-C_12_-LAB	19.5	16.6	36.5	41.2	23.3	54.6	63.6	47.9	70.3	42.0	55.1	29.9	41.9	53.4	37.6	31.3	48.4
5-C_12_-LAB	17.9	15.6	33.9	38.4	21.9	50.8	58.9	46.4	66.2	39.3	51.3	28.6	38.6	50.4	35.5	29.6	45.4
4-C_12_-LAB	12.8	11.8	24.9	28.3	14.7	36.9	41.6	36.7	48.3	29.0	39.4	20.6	26.7	35.6	28.0	22.2	33.3
3-C_12_-LAB	8.6	8.6	16.9	20.6	10.5	25.7	29.9	31.4	34.8	20.1	25.8	16.8	20.5	25.4	21.2	15.4	22.8
2-C_12_-LAB	9.7	15.8	22.8	26.5	15.5	35.6	42.1	76.8	46.2	26.2	36.8	30.6	25.3	36.0	41.9	23.2	28.2
7-C_13_-LAB	8.1	12.5	18.3	18.1	11.6	25.5	30.9	55.8	33.0	19.6	29.3	22.7	20.4	28.1	30.6	17.9	19.4
6-C_13_-LAB	8.1	12.5	18.3	18.1	11.6	25.5	30.9	55.8	33.0	19.6	29.3	22.7	20.4	28.1	30.6	17.9	19.4
5-C_13_-LAB	9.8	15.6	22.9	22.8	14.0	30.5	38.6	69.2	41.5	24.8	35.7	27.0	25.2	35.1	37.6	22.0	24.7
4-C_13_-LAB	7.3	11.6	16.6	16.7	11.0	22.9	28.9	57.0	29.7	18.2	26.2	21.5	18.6	25.7	29.5	17.1	16.8
3-C_13_-LAB	5.7	10.1	11.3	13.3	8.6	16.3	19.0	43.9	21.6	14.5	20.3	14.5	14.0	19.1	21.5	13.4	12.4
2-C_13_-LAB	15.5	33.6	36.1	38.6	27.9	49.3	60.6	175.1	56.7	41.1	57.6	59.7	35.2	56.6	85.7	41.0	31.4
7-C_14_-LAB	<1.05	<1.05	<1.05	<1.05	<1.05	<1.05	<1.05	<1.05	<1.05	<1.05	<1.05	<1.05	<1.05	<1.05	<1.05	<1.05	<1.05
6-C_14_-LAB	<1.05	<1.05	<1.05	<1.05	<1.05	<1.05	<1.05	<1.05	<1.05	<1.05	<1.05	<1.05	<1.05	<1.05	<1.05	<1.05	<1.05
5-C_14_-LAB	<1.05	<1.05	<1.05	<1.05	<1.05	<1.05	<1.05	<1.05	<1.05	<1.05	<1.05	<1.05	<1.05	<1.05	<1.05	<1.05	<1.05
4-C_14_-LAB	<1.05	<1.05	<1.05	<1.05	<1.05	<1.05	<1.05	<1.05	<1.05	<1.05	<1.05	<1.05	<1.05	<1.05	<1.05	<1.05	<1.05
3-C_14_-LAB	<1.05	<1.05	<1.05	<1.05	<1.05	<1.05	<1.05	<1.05	<1.05	<1.05	<1.05	<1.05	<1.05	<1.05	<1.05	<1.05	<1.05
2-C_14_-LAB	<1.05	<1.05	<1.05	<1.05	<1.05	<1.05	<1.05	<1.05	<1.05	<1.05	<1.05	<1.05	<1.05	<1.05	<1.05	<1.05	<1.05

**Table 9:** Concentrations of Linear alkylbenzenes (LABs) in whole-body tissues of *Anomalocardia flexuosa* exposed to sediments from Mucuripe Bay and reference site located at Icapuí (Survey 2). Values expressed in ng g^−1^ (dry weight).

Sample	Control	Reference				MD1				MD2					MD3		
Exposure time	Day 0	Day 7	Day 14	Day 21	Day 28	Day 7	Day 14	Day 21	Day 28	Day 7	Day 14	Day 21	Day 28	Day 7	Day 14	Day 21	Day 28
5-C_10_ LAB	17.2	23.0	27.5	17.0	13.0	22.0	25.9	32.3	41.7	19.1	23.3	26.8	34.6	18.4	20.7	39.7	25.8
4-C_10_-LAB	13.4	18.6	22.3	13.9	10.5	18.6	21.6	27.8	35.9	15.9	20.2	22.7	29.4	16.0	17.3	33.1	22.4
3-C_10_-LAB	11.1	15.7	18.4	11.9	9.3	15.7	20.1	23.4	30.7	14.4	16.3	20.1	26.2	13.4	15.1	27.9	19.7
2-C_10_-LAB	13.7	20.7	22.1	16.9	12.7	24.1	33.3	34.7	48.3	23.3	25.5	30.0	42.5	19.4	21.3	37.2	31.2
6-C_11_-LAB	18.0	29.1	28.5	26.0	17.8	34.1	47.7	53.1	71.9	28.7	37.2	45.7	60.5	27.2	34.7	46.6	47.3
5-C_11_-LAB	22.4	51.4	43.0	41.7	25.7	55.5	78.8	91.0	136.5	50.7	65.0	78.1	107.1	44.5	54.0	85.1	80.8
4-C_11_-LAB	16.5	38.5	30.9	31.6	19.0	42.8	60.1	68.6	102.2	38.4	49.8	58.4	81.8	33.5	41.8	61.5	61.6
3-C_11_-LAB	11.2	27.7	21.9	23.3	13.7	33.4	43.6	49.0	70.7	28.0	37.0	43.7	60.6	24.1	30.2	44.1	45.2
2-C_11_-LAB	13.3	34.6	22.5	29.9	22.1	43.8	54.5	56.3	80.5	33.3	47.1	48.7	74.3	28.8	38.4	48.7	53.1
6-C_12_-LAB	14.5	41.8	22.8	35.8	26.3	55.4	59.3	65.1	111.7	40.9	53.9	56.8	85.2	32.5	45.8	52.4	61.8
5-C_12_-LAB	12.5	39.2	21.2	34.1	26.5	53.1	55.5	61.4	101.9	37.6	50.5	53.5	78.6	30.3	43.3	48.7	57.7
4-C_12_-LAB	8.8	29.5	15.5	24.6	21.1	39.0	40.4	43.2	71.3	26.1	36.8	37.9	57.5	22.4	29.3	34.0	41.5
3-C_12_-LAB	7.1	20.1	10.4	17.7	14.4	27.6	26.7	29.9	46.1	18.2	26.4	25.3	37.8	15.4	23.5	23.7	27.1
2-C_12_-LAB	12.9	27.1	15.6	22.6	23.2	34.8	32.5	33.9	46.5	21.3	31.3	28.8	45.2	21.1	27.2	28.3	31.4
7-C_13_-LAB	8.7	19.4	11.6	18.5	16.8	25.0	26.0	29.3	34.9	14.9	25.0	24.9	36.7	16.5	22.8	23.5	27.3
6-C_13_-LAB	8.7	19.4	11.6	18.5	16.8	25.0	26.0	29.3	34.9	14.9	25.0	24.9	36.7	16.5	22.8	23.5	27.3
5-C_13_-LAB	11.7	24.3	13.8	22.4	20.8	30.6	31.4	35.5	42.5	18.7	30.3	29.9	44.6	20.2	27.5	29.0	33.0
4-C_13_-LAB	8.7	18.2	11.5	16.2	16.6	21.2	23.2	25.7	29.2	13.3	22.2	21.3	32.1	15.9	19.9	20.8	23.8
3-C_13_-LAB	8.6	15.0	10.6	13.7	13.5	21.4	18.9	20.3	23.3	10.6	18.2	18.5	26.2	12.3	17.1	17.9	20.7
2-C_13_-LAB	28.6	40.3	33.9	34.0	40.7	45.2	41.9	45.6	44.6	28.3	38.8	39.5	52.0	36.9	43.7	44.0	40.8
7-C_14_-LAB	<1.05	<1.05	<1.05	<1.05	<1.05	<1.05	<1.05	<1.05	<1.05	<1.05	<1.05	<1.05	<1.05	<1.05	<1.05	<1.05	<1.05
6-C_14_-LAB	<1.05	<1.05	<1.05	<1.05	<1.05	<1.05	<1.05	<1.05	<1.05	<1.05	<1.05	<1.05	<1.05	<1.05	<1.05	<1.05	<1.05
5-C_14_-LAB	<1.05	<1.05	<1.05	<1.05	<1.05	<1.05	<1.05	<1.05	<1.05	<1.05	<1.05	<1.05	<1.05	<1.05	<1.05	<1.05	<1.05
4-C_14_-LAB	<1.05	<1.05	<1.05	<1.05	<1.05	<1.05	<1.05	<1.05	<1.05	<1.05	<1.05	<1.05	<1.05	<1.05	<1.05	<1.05	<1.05
3-C_14_-LAB	<1.05	<1.05	<1.05	<1.05	<1.05	<1.05	<1.05	<1.05	<1.05	<1.05	<1.05	<1.05	<1.05	<1.05	<1.05	<1.05	<1.05
2-C_14_-LAB	<1.05	<1.05	<1.05	<1.05	<1.05	<1.05	<1.05	<1.05	<1.05	<1.05	<1.05	<1.05	<1.05	<1.05	<1.05	<1.05	<1.05

## 2 Experimental design, materials and methods

The sediment sampling was conducted on January 24, 2011, during the intense dredging of Mucuripe bay (survey 1), and on July 29, 2011, at the end of dredging activities (survey 2). Samples were taken using a *van* Veen grab sampler (0.026 m^2^). Aliquots for toxicity tests were stored in the laboratory at 4 °C. Another aliquot was wrapped into pre-cleaned aluminum foil and stored at −20 °C for the analysis of hydrocarbons. Clams of the species *Anomalocardia flexuosa* were collected in sediment banks, in the city of Icapuí (Ceará state), at Requenguela beach during the low tide. Animals were kept in thermal boxes, transferred to the laboratory, and acclimated to clean seawater (25 °C and salinity of 35 ‰) for 10 days prior to experiments.

Sediment toxicity test with *A. flexuosa* was performed in triplicate per site in 5 L glass bottles containing 500 g of whole-sediment sample and 5 L of clean and filtered seawater (45 µm, salinity 35‰). After 24 h of equilibration period, 7 healthy organisms (juveniles, 15 mm length) were introduced into each bottle and the exposure system was kept under photoperiod (12 h light: 12 h dark), with constant aeration and temperature (25 ± 2 °C). The experiment lasted 28 days, and four batches were prepared for each 7 days intervals (7, 14, 21, and 28). No mortality of clams was observed and at each time of exposure the system was ended, and the animals euthanized by ice-based method for the sampling of whole-body soft tissues. Tissues from 8 animals were pooled and freeze-dried for the determinations of hydrocarbons analysis.

Sediment analysis consisted of hydrocarbons determination of freeze-dried samples, conducted in September 2012: aliphatic (AHs: 26 compounds), polycyclic aromatic hydrocarbons (PAHs; 39 compounds), and linear alkylbenzenes (LABs; 26 compounds) [2]. Whole-body soft tissues were analyzed for the same compounds with procedures described in a previous study [3]. Then, AHs were quantified on a gas chromatography (GC) model 6890 from Agilent Technologies with flame ionization detector (GC-FID), while PAHs and LABs were quantified on GC coupled to a 5973 N mass spectrometer (GC-MS) in a selected ion mode (SIM).

## 3 Ethics statement

Experiments performed complies with the ARRIVE guidelines.

## Declaration of Competing Interest

The authors declare that they have no known competing financial interests or personal relationships which have, or could be perceived to have, influenced the work reported in this article.
